# Differential sleep-promoting effects of dual orexin receptor antagonists and GABA_A_ receptor modulators

**DOI:** 10.1186/1471-2202-15-109

**Published:** 2014-09-22

**Authors:** Anthony L Gotter, Susan L Garson, Joanne Stevens, Regina L Munden, Steven V Fox, Pamela L Tannenbaum, Lihang Yao, Scott D Kuduk, Terrence McDonald, Jason M Uslaner, Spencer J Tye, Paul J Coleman, Christopher J Winrow, John J Renger

**Affiliations:** Department of Neuroscience, Merck Research Laboratories, 770 Sumneytown Pike, PO Box 4, West Point, PA 19486-0004 USA; Department of In Vivo Pharmacology, Merck Research Laboratories, 770 Sumneytown Pike, PO Box 4, West Point, PA 19486-0004 USA; Department of Medicinal Chemistry, Merck Research Laboratories, 770 Sumneytown Pike, PO Box 4, West Point, PA 19486-0004 USA; Department of Comparative Medicine, Penn State University College of Medicine, Hershey, PA USA; Novira Therapeutics, Doylestown, PA USA

**Keywords:** Orexin, Hypocretin, Benzodiazepine, Insomnia, Sleep, Electroencephalography, Sleep deprivation, REM sleep, NREM sleep, Suvorexant, Belsomra

## Abstract

**Background:**

The current standard of care for insomnia includes gamma-aminobutyric acid receptor A (GABA_A_) activators, which promote sleep as well as general central nervous system depression. Dual orexin receptor antagonists (DORAs) represent an alternative mechanism for insomnia treatment that induces somnolence by blocking the wake-promoting effects of orexin neuropeptides. The current study compares the role and interdependence of these two mechanisms on their ability to influence sleep architecture and quantitative electroencephalography (qEEG) spectral profiles across preclinical species.

**Results:**

Active-phase dosing of DORA-22 induced consistent effects on sleep architecture in mice, rats, dogs, and rhesus monkeys; attenuation of active wake was accompanied by increases in both non─rapid eye movement (NREM) and rapid eye movement (REM) sleep. Eszopiclone, a representative GABA_A_ receptor modulator, promoted sleep in rats and rhesus monkeys that was marked by REM sleep suppression, but had inconsistent effects in mice and paradoxically promoted wakefulness in dogs. Active-phase treatment of rats with DORA-12 similarly promoted NREM and REM sleep to magnitudes nearly identical to those seen during normal resting-phase sleep following vehicle treatment, whereas eszopiclone suppressed REM even to levels below those seen during the active phase. The qEEG changes induced by DORA-12 in rats also resembled normal resting-phase patterns, whereas eszopiclone induced changes distinct from normal active- or inactive-phase spectra. Co-dosing experiments, as well as studies in transgenic rats lacking orexin neurons, indicated partial overlap in the mechanism of sleep promotion by orexin and GABA modulation with the exception of the REM suppression exclusive to GABA_A_ receptor modulation. Following REM deprivation in mice, eszopiclone further suppressed REM sleep while DORA-22 facilitated recovery including increased REM sleep.

**Conclusion:**

DORAs promote NREM and importantly REM sleep that is similar in proportion and magnitude to that seen during the normal resting phase across mammalian animal models. While limited overlap exists between therapeutic mechanisms, orexin signaling does not appear involved in the REM suppression exhibited by GABA_A_ receptor modulators. The ability of DORAs to promote proportional NREM and REM sleep following sleep deprivation suggests that this mechanism may be effective in alleviating recovery from sleep disturbance.

**Electronic supplementary material:**

The online version of this article (doi:10.1186/1471-2202-15-109) contains supplementary material, which is available to authorized users.

## Background

Currently, most pharmacologic treatments for insomnia are central nervous system depressants that act by allosterically activating gamma-aminobutyric acid receptor A (GABA_A_) [1–4]. More recently, the inhibition of orexin-mediated arousal has attracted interest as a potential mechanism for treating insomnia [5, 6]. Orexin neurons, which are active during wakefulness but quiescent during sleep [7], are localized in the lateral hypothalamus and project to multiple brain regions, including regions involved with regulating sleep and wakefulness [8]. Several orexin receptor antagonists (ORAs) have been developed to interfere with orexin signaling by blocking one or both orexin receptors (OX_1_R and OX_2_R) and have demonstrated the ability to safely promote sleep in preclinical models and clinical trials [9–12].

Emerging preclinical and clinical data have suggested that GABA_A_ receptor modulators and ORAs may differentially impact sleep parameters, particularly with regard to rapid eye movement (REM) sleep. Studies in rats have demonstrated that the GABA_A_ receptor modulators eszopiclone and zolpidem disproportionately promote non─rapid eye movement (NREM) sleep while suppressing REM sleep. On the other hand, dual orexin receptor antagonists (DORAs) are known to increase both REM and NREM sleep [10, 13]. Similarly, in human subjects with situational insomnia, both zolpidem and the DORA SB-649868 increased total sleep time; SB-649868 reduced REM latency and increased duration compared with placebo, and zolpidem significantly reduced the proportion of the sleep period spent in REM sleep [14]. Eszopiclone and zolpidem also dose-dependently disrupted sleep-stage─specific electroencephalography (EEG) spectral profiles in rats, even at low doses that do not induce sleep. In contrast, only the highest dose of DORA-22 tested (30 mg/kg) had marginal effects on EEG power spectral frequency, and these effects were observed only during REM sleep [13]. No significant differences in EEG spectra were observed in clinical trials with suvorexant (Belsomra®) compared with placebo in both healthy subjects [15] and in patients with insomnia [16, 17]. Zolpidem, but not SB-649868, induced EEG disruptions in subjects with situational insomnia [14]. The impact of the observed alterations in sleep architecture with GABA_A_ receptor modulators, particularly on REM sleep, remains to be determined.

Chronic total and REM sleep deprivation in rats leads to significant morbidity and mortality [18]. Even acute sleep restriction, particularly the suppression of REM sleep, impacts memory consolidation in preclinical behavioral models [19, 20]. In humans, anecdotal and clinical evidence suggests that total sleep deprivation may be associated with cognitive impairments (as reviewed in Banks and Dinges, 2007, and Basner et al., 2013 [21, 22]). The impact of selective REM deprivation in humans is less clear, but effects on memory consolidation and pain perception have been reported [23, 24].

The present study compared sleep induced by the standard of care, eszopiclone (a non-benzodiazepine GABA_A_ receptor modulator) and by two distinct DORAs, DORA-12 and DORA-22, using polysomnography (PSG) and quantitative electroencephalography (qEEG) spectral analysis following dose administration during the active phase across several species and following REM deprivation in rats. Direct comparisons with sleep architecture seen during the normal resting phase revealed that DORAs proportionately induced both NREM and REM sleep in a pattern no different from non-medicated sleep and induced qEEG changes consistent with those seen during the inactive phase. Eszopiclone had differential effects on sleep across species that were characterized by abnormal sleep architecture and qEEG profiles relative to vehicle-treated, inactive-phase control animals. Further, DORAs more immediately facilitated proportional recovery sleep following REM deprivation relative to vehicle or eszopiclone treatment. Co-dosing studies and experiments in orexin/ataxin-3 (*Ox/Atx*) transgenic rats lacking orexin neurons indicated only partial overlap of these two mechanisms.

## Results

### DORAs and standard of care differentially affect sleep across mammalian species

**Figure 1:**
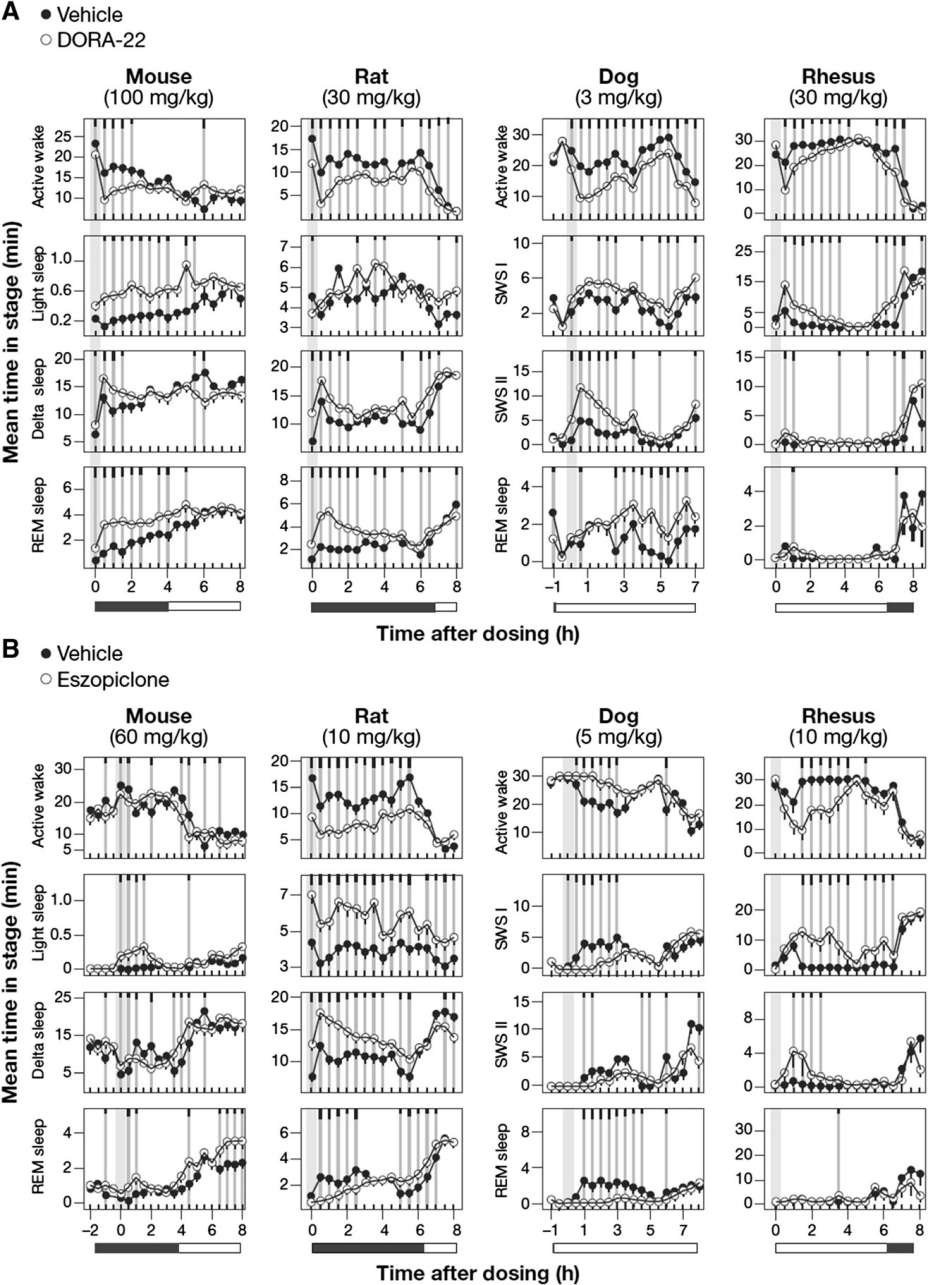
**Sleep architecture responses to DORA-22 and eszopiclone across species.** The mean time spent in each sleep stage during 30-min intervals is plotted for compound (open circles) and vehicle (closed circles) conditions. **A**. Sleep architecture following DORA-22 treatment relative to vehicle (vitamin E TPGS [d-alpha tocopheryl polyethylene glycol 1000 succinate], 20% solution, orally) in mice (100 mg/kg, n = 11), rats (30 mg/kg, n = 13), dogs (3 mg/kg, n = 6), and rhesus monkeys (30 mg/kg, n = 6). SWS I and SWS II refers to lighter NREM I sleep and NREM II sleep that includes a preponderance of delta qEEG power, respectively. **B**. Sleep architecture following eszopiclone treatment relative to vehicle (vitamin E TPGS, 20% solution, orally) in mice (60 mg/kg, n = 6), rats (10 mg/kg, n = 16), dogs (5 mg/kg, n = 6), and rhesus monkeys (10 mg/kg, n = 6). Time of dose indicated by gray bars. Mean times in each vigilance state during 30-min intervals by condition were averaged over all days of treatment from a crossover design such that all subjects received each treatment as described in Methods. Significant differences from vehicle are indicated by gray vertical lines, with black tic marks indicating significance level (short, medium, long, P < 0.05, 0.01, 0.001; linear mixed-effects model for repeated measures t-test).

To qualitatively compare the sleep architecture induced by orexin receptor antagonism versus GABA_A_ receptor modulation across species, PSG analyses were conducted in telemetry-implanted mice, rats, dogs, and rhesus monkeys receiving either DORA-22 or eszopiclone during their usual active period, and evaluated relative to vehicle treatment. Representative doses for each species were chosen based on prior dose response studies inducing salient, active-wake reduction approximating that seen in rats, the highest dose tested (DORA-22 in rhesus monkeys), or a qEEG effect magnitude similar to that in other species (eszopiclone in mice, dogs). In all four species examined, significant active-wake reduction at consecutive 30 min time points by DORA-22 ranged from 2 h (mice) to 7 h (dogs), and was associated with significant increases in both NREM and REM sleep (Figure 1A). In mice and rats, delta sleep showed immediate 2 and 2.5 hour increases (P ≤ 0.05 at four and five consecutive 30 min time points), respectively, while REM sleep was significantly increased for 3 and up to 5.5 h, respectively. Variable increases in light sleep, comprising the smallest proportion of vigilance state mean time, were also observed in mice and to a lesser extent in rats. In dogs and rhesus monkeys, NREM I sleep was similarly increased with a delay in dogs at time points up to 7 h after dosing and for 5.5 h in rhesus immediately after dosing coincident with significant decreases in active wake at time points up to 7 h following treatment. NREM II was increased in both animals with dogs showing a greater magnitude change (4.5 h in dogs; 1 hour in monkeys), whereas increases in REM increases were delayed in both animals (4.5 h in dogs; 30 min in rhesus) relative to that seen in rodents, likely due to the slower sleep cycle times in these higher species. The decreased magnitude of effects seen in the rhesus monkeys relative to other species is a result of lower compound exposure levels for this compound and not the orexin receptor antagonist mechanism. The maximum plasma concentration (C_max_) following 30 mg/kg administration of DORA-22 to these animals reached 0.14 μM compared to 1.42 μM in rats (30 mg/kg), 3.36 μM in mice (100 mg/kg) and 1.62 μM in dogs (3 mg/kg) in these experiments.

Eszopiclone, on the other hand, had inconsistent effects on sleep architecture across species. Attenuation of active wake was observed in rats (significant time points [p ≤ 0.05] for 6 h), rhesus monkeys (3 h), and variably in mice, but paradoxically was associated with increased arousal in dogs (3 h of consecutive time points) (Figure 1B). In those animals in which sleep was induced, compound-dependent increases were observed in both light sleep (up to 8 h in rats; 6.5 h in rhesus) and delta or NREM II sleep (up to 5.5 h in rat; 2 h in Rhesus), while mice showed increases in light sleep (P ≤ 0.05 for 2 h), but variable decreases in delta sleep. Immediate suppression of REM sleep was common in rats (2.5 h) and dogs (4 h), but was not observed in mice. Rhesus monkeys showed no compound-dependent REM decreases, but minimal baseline levels during the active phase likely precluded any detectable decrease in these animals. The increased arousal observed in dogs was consistent with that previously observed at different doses [25] and similar to that observed for other benzodiazepines [26–28]. As might be expected, increases in active wake were associated with decreases in both NREM and REM sleep in these animals. Together these results demonstrate variable sleep responses to eszopiclone across species, but also identify rats as the preclinical species in which the sleep-promoting effects for both classes of drugs can be reliably demonstrated.

### DORA-induced REM and NREM sleep mimics normal resting-phase sleep

**Figure 2:**
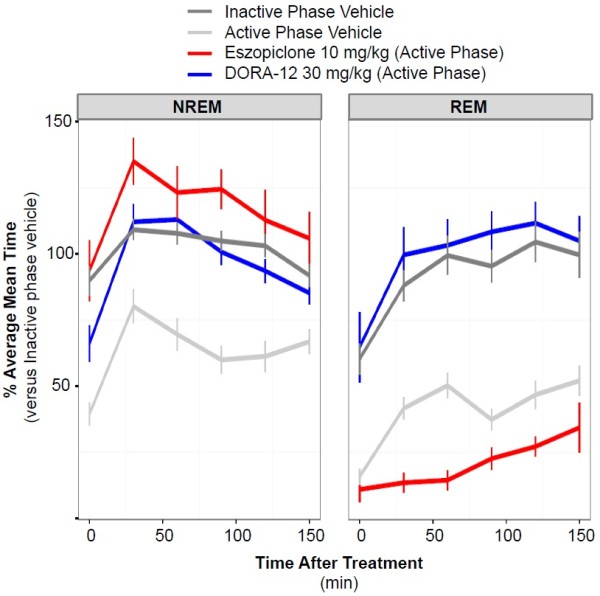
**Sleep architecture induced by DORA-12 is no different from normal resting-phase sleep in rats.** The time course and magnitude of mean time spent in NREM and REM sleep in 30-min intervals following active-phase treatment with DORA-12 (30 mg/kg, n = 14) and eszopiclone (10 mg/kg, n = 16) is compared with that following vehicle treatment (vitamin E TPGS, 20% solution, orally) at both the onset of the active phase (Zeitgeber time [ZT] 17:30) and the inactive phase (ZT 00:00). Values at 30-min intervals are expressed as percentage of the mean 30-min level calculated from times 90 to 150 min of the inactive-phase onset levels. Data represent a 3-h summary of the full time course and polysomnographic analysis presented in Additional file 1: Figure S1; light and delta sleep have been combined as NREM sleep. Comparison of treatment conditions analyzed by analysis of variance (ANOVA) followed by the Tukey multiple comparison (HSD) test revealed significant differences (P < 0.0001) between all conditions except the following: inactive-phase onset NREM vs DORA-12 NREM (P = 0.0661), eszopiclone NREM vs DORA-12 NREM (P = 0.2582), and inactive-phase onset REM vs DORA-12 REM (P = 0.2933). Similar results were seen when each condition was evaluated pairwise by 2-way ANOVA: eszopiclone vs DORA-12 (NREM, F_1,10_ = 7.43, P = 0.0214; REM, F_1,10_ = 120.9, P < 0.0001); active phase vehicle vs DORA-12 (NREM, F_1,10_ = 26.67, P < 0.0001; REM, F_1,10_ = 183.4, P < 0.0001); inactive phase vehicle vs DORA-12 (NREM, F_1,10_ = 0.227, P = 0.644, REM, F_1,10_ = 2.347, P = 0.157); active phase vehicle vs eszopiclone (NREM, F_1,10_ = 76.72, P < 0.0001; REM, F_1,10_ = 8.86, P = 0.0139); inactive phase vehicle vs eszopiclone (NREM, F_1,10_ = 7.68, P = 0.0197; REM, F_1,10_ = 51.92, P < 0.0001); inactive phase vehicle vs active phase vehicle (NREM, F_1,10_ = 56.57, P < 0.0001; REM, F_1,10_ = 41.19, P < 0.0001).

To determine how the sleep architecture induced by both classes of drugs compares with that seen during the normal resting phase, we compared the effects of DORA-12 and eszopiclone administered during the active phase to the NREM and REM sleep seen normally during the inactive phase after vehicle dosing in rats. A DORA of distinct chemical structure, DORA-12 was used herein to further substantiate the observed effects of DORA-22 on general inactive-phase sleep effects and qEEG in a prior sleep-stage analysis [13]. Figure 2 directly compares the NREM and REM changes occurring during the normal inactive phase with those seen during the active phase by shifting the data collected during the normal inactive-phase onset of 6.5 h to coincide with the active-phase vehicle treatment time (Zeitgeber time [ZT] 17:30; see also Additional file 1: Figure S1A). As expected, the mean time spent in NREM and REM following vehicle treatment during the active phase was significantly lower than that seen during the 150 min following the onset of the vehicle treatment inactive phase (NREM: F_1,10_ = 56.57, P < 0.0001, 2-way ANOVA, P < 0.0001, Tukey HSD; REM: F_1,10_ = 41.19, P < 0.0001, 2-way ANOVA, P < 0.0001, Tukey HSD). Following active-phase treatment with eszopiclone (10 mg/kg), the time course of NREM was significantly increased relative to both active-phase vehicle (F_1,10_ = 76.72, P < 0.0001, 2-way ANOVA; P < 0.0001, Tukey HSD) and that occurring during the onset of the inactive phase (F_1,10_ = 7.68, P = 0.0197, 2-way ANOVA; P < 0.0001, Tukey HSD), whereas REM sleep was significantly decreased relative to both control conditions (active phase: F_1,10_ = 8.86, P = 0.0139, 2-way ANOVA; P < 0.0001, Tukey HSD; inactive phase: F_1,10_ = 51.92, P < 0.0001, 2-way ANOVA; P < 0.0001, Tukey HSD). On the other hand, treatment with DORA-12 (30 mg/kg) during the active phase increased both NREM and REM sleep similar to that observed following vehicle administration during the inactive phase onset. Remarkably, the time courses of these increases were statistically no different from those observed during the inactive-phase onset following vehicle treatment (NREM: F_1,10_ = 0.227, P = 0.644, 2-way ANOVA, P = 0.0661; Tukey HSD; REM: F_1,10_ = 2.347, P = 0.157, 2-way ANOVA, P = 0.2933, Tukey HSD). A comprehensive comparison of the time courses of vigilance states with vehicle, DORA-12, and eszopiclone treatments during both the active phase and inactive phase is shown in Additional file 1: Figure S1; eszopiclone suppressed REM sleep while promoting NREM sleep in a manner distinct from that seen during the normal resting phase, whereas DORA-12 induced minimal changes relative to normal, inactive-phase sleep.

**Figure 3:**
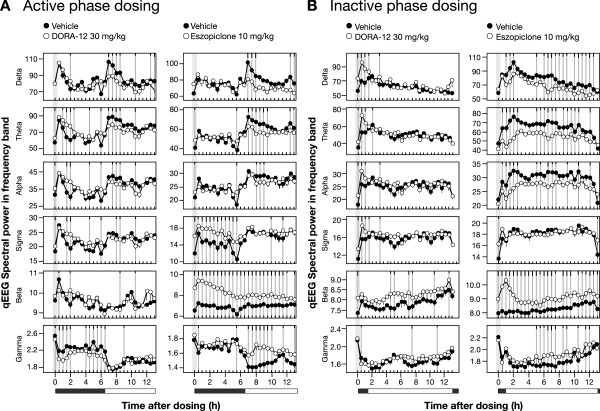
**The qEEG spectral pattern induced by DORA-12 in rats mimics the inactive phase.** Mean qEEG spectral power in indicated frequency bands at 30-min analysis intervals following treatment with DORA-12and eszopiclone (open symbols) was evaluated relative to vehicle (vitamin E TPGS, 20% solution, orally, closed symbols), coincident with PSG analysis. **A**. Active-phase (ZT 17:30) treatment with DORA-12 (30 mg/kg, n = 14) and eszopiclone (10 mg/kg, n = 16). **B**. Treatment 1 h prior to the inactive-phase onset (ZT 23:00) with DORA-12 (30 mg/kg, n = 7) and eszopiclone (10 mg/kg, n = 8). Gray vertical bars represent dose time. Significant differences from vehicle are indicated by gray vertical lines; black tic marks indicate significance level (short, medium, long, P < 0.05, 0.01, 0.001; linear mixed-effects model for repeated measures t-test).

Quantitative EEG analysis in these same rats following treatment with DORA-12 (30 mg/kg) and eszopiclone (10 mg/kg) during both the active phase and at the onset of the resting phase further illustrates differences between these two mechanisms. DORA-12 administration during the active phase was associated with time-dependent decreases in high-frequency gamma power (30–100 Hz) (significant time points [P ≤ 0.05] up to 6.5 h following dosing), increases in middle-frequency spectral power (theta up to 3 h; alpha, 2.5 h; sigma, 2 h), and little change relative to vehicle in delta frequency (0.5–4 Hz) (Figure 3A). These changes largely dissipated 6.5 h later with the onset of the inactive phase, where DORA-12 responses approached those of vehicle-treated animals. In contrast, eszopiclone treatment during the active phase resulted in large increases in qEEG power in the beta band (19–30 Hz) (12 h) as well as sigma frequencies (12–16 Hz) (5.5 h) whereas minimal decreases were seen in theta and alpha power (4–7 Hz and 8–12 Hz, respectively). Relative to vehicle, eszopiclone induced little-to-no change in gamma and delta powers until the onset of the inactive phase 6.5 h later, when levels of gamma, delta, and theta power were maintained even though vehicle-treated animals exhibited substantial decreases in gamma power and increases in delta and theta power associated with sleep onset. These inactive-phase differences induced by eszopiclone were corroborated when the drug was administered 1 h prior to the inactive-phase onset, where substantial changes relative to the vehicle condition were seen in most frequency bands (Figure 3B). DORA-12, on the other hand, induced shorter-duration decreases in gamma frequencies and increases in low-to-mid frequency powers, changes that exaggerated the normal change displayed in the vehicle condition during the resting period. Overall, both DORA-12 and eszopiclone appeared to have similar effects on qEEG power during both the active and inactive phases, but their observed effects relative to vehicle were dependent on baseline changes occurring at different times of day. Relative to vehicle, DORAs appeared to have less effect on qEEG power during the inactive phase, since baseline levels already reflected these changes during the normal resting period.

### DORA-22 promotes recovery from REM sleep deprivation

**Figure 4:**
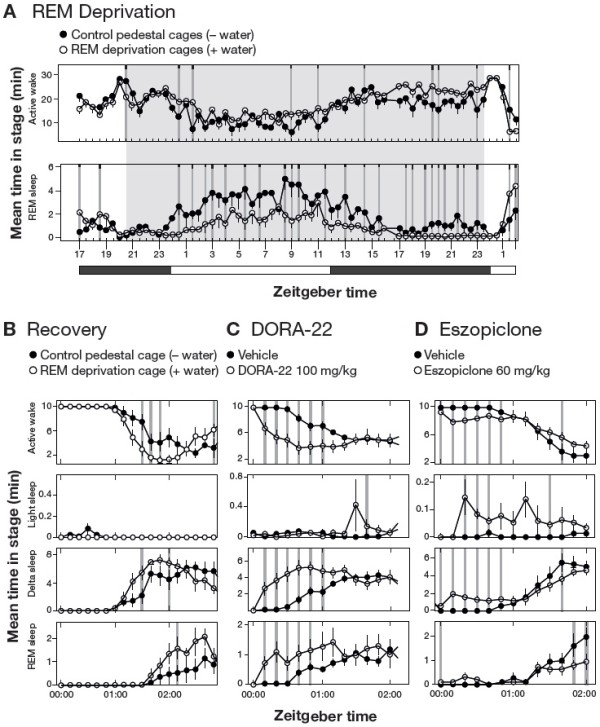
**DORA-22 facilitates REM recovery following REM deprivation. A**. REM deprivation in mice utilizing the pedestal method for 28 h (ZT 20:00 to ZT 24:00/00:00 on the subsequent day, n = 8 wild-type C57/BL6 mice in a balanced 2 × 1 day crossover). **B**. Sleep architecture at the cessation of 28 h of REM deprivation. Mice were alternately housed in cages containing pedestals and either no water (control condition, closed symbols) or water (REM deprivation condition, open symbols) in a balanced 2 × 1 day crossover as described in Methods (n = 8 wild-type C57/BL6 mice). Time spent in the indicated sleep stages immediately upon cage change to normal bedding (ZT 00:00, or lights-on) is shown. **C**. Effects of DORA-22 treatment (100 mg/kg p.o., open symbols) relative to vehicle (vitamin E TPGS, 20% solution, orally, closed symbols) on sleep architecture when administered coincident with the cessation of REM deprivation (ZT 00:00, n = 11 wild-type C57/BL6 mice in a balanced 2 × 1 day crossover in which each animal alternately received vehicle and DORA-22). **D**. Effects of eszopiclone treatment (60 mg/kg p.o., open symbols) relative to vehicle (vitamin E TPGS, 20% solution, orally, closed symbols) coincident with the cessation of REM deprivation (ZT 00:00, n = 11 wild-type C57/BL6 mice in a balanced 2 × 1 day crossover in which each animal alternately received vehicle and eszopiclone). Significant differences are indicated by gray vertical lines, and black tic marks indicate significance level (short, medium, long, P < 0.05, 0.01, 0.001).

Given the ability of DORA-22 to promote somnolence including REM sleep and, conversely, the potential for eszopiclone to promote sleep associated with REM suppression, we evaluated the ability of both compounds to promote recovery from REM sleep deprivation. For these experiments, mice were first subjected to 28 h of REM deprivation using the platform or "flower pot" paradigm [29], followed immediately thereafter by administration of vehicle (vitamin E TPGS [d-alpha tocopheryl polyethylene glycol 1000 succinate], 20% solution, p.o.), DORA-22 (100 mg/kg) or eszopiclone (60 mg/kg), or no treatment. This manipulation involves placing mice in cages containing 3-cm-diameter platforms surrounded by water (REM deprivation condition), such that the atonia accompanying REM sleep is associated with the animals slipping into the water and arousing. In initial control experiments to demonstrate the feasibility of this approach, mice in cages containing pedestals with water were compared with mice housed similarly, but in the absence of water. As expected, mice in the REM deprivation condition exhibited significant reductions in the amount of time spent in REM sleep at time points throughout the duration of the manipulation (Figure 4A). Upon transfer to cages containing normal bedding at ZT 0:00, a condition representing a novel environment, both control and REM-deprived animals exhibited maximal active wake for approximately 1 h (Figure 4B). Shortly thereafter, mean time in active wake began to decrease in both groups with the REM-deprived group exhibiting significantly less active wake at 3 time points, and increased delta sleep at early time points. Similarly, REM sleep also increased in both groups with the REM deprivation group exhibiting more REM recovery relative to those in the control condition. The administration of DORA-22 occurred simultaneously with the transfer of animals to normal conditions and induced immediate active-wake attenuation coincident with increases in both delta and REM sleep, relative to vehicle-treated animals (significant changes at 1 hour and 50 min relative to vehicle) (Figure 4C). Unlike the changes seen during normal recovery, these changes occurred within 10 min after transfer to cages containing normal bedding. Eszopiclone also facilitated active-wake reductions for 1 hour immediately upon return to normal cages following REM deprivation (Figure 4D). However, in contrast to DORA-22 treatment, this manipulation was associated with increases in light and delta sleep at early time points with little change in REM sleep. Even in what was expected to be increased REM pressure, this GABA_A_ receptor modulator significantly suppressed REM at later time points relative to vehicle-treated animals.

### GABA_A_ modulators suppress REM independently of orexin signaling

**Figure 5:**
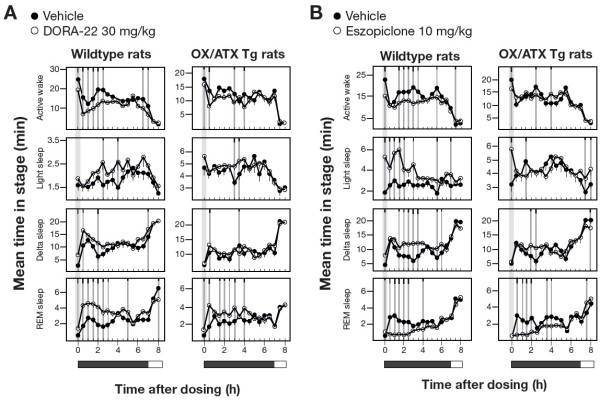
**Sleep architecture induced by DORA-22 and eszopiclone in wild-type and** ***OX/Atx*** **transgenic rats. A**. PSG analysis of DORA-22 (30 mg/kg) relative to vehicle (vitamin E TPGS, 20% solution, orally) in wild-type (n = 7) and *Ox/Atx* (n = 7) Sprague–Dawley rats. **B**. PSG analysis of eszopiclone (10 mg/kg) relative to vehicle (vitamin E TPGS, 20% solution, orally) in wild-type (n = 7) and *Ox/Atx* (n = 7) Sprague–Dawley rats. Significant differences are indicated by gray vertical lines, and black tic marks indicate significance level (short, medium, long, P < 0.05, 0.01, 0.001).

To determine the interaction between GABA_A_ receptor modulator and DORA-mediated mechanisms, and the dependence of eszopiclone activity on orexin signaling, we first evaluated the effectiveness of both DORA-22 (30 mg/kg) and eszopiclone (10 mg/kg) in *Ox/Atx* transgenic rats deficient in orexin neurons (expressing the cytotoxic *poly-Q-ataxin-3* gene product via the *HCRT* (*hypocretin*) gene promoter [30]). Relative to wild-type Sprague–Dawley rats evaluated simultaneously, DORA-22 resulted in diminished, but still detectable responses in *Ox/Atx* transgenic rats (Figure 5A), consistent with the diminished, yet incomplete, ablation of orexin signaling in this model (the selectivity of DORA-22 at 100 mg/kg has previously been demonstrated in mice lacking both OX_1_R and OX_2_R [12]). The sleep-promoting effects of eszopiclone were also diminished in *Ox/Atx* transgenic rats relative to the effects seen in wild-type Sprague–Dawley rats (Figure 5B), the exception being in the magnitude of REM reduction where significant reductions were seen up to 4 h post administration. Together these results suggest that eszopiclone mediates many of its sleep-promoting responses through orexin signaling with the exception of its effects on REM, which persist in the presence of reduced orexin signaling.

**Figure 6:**
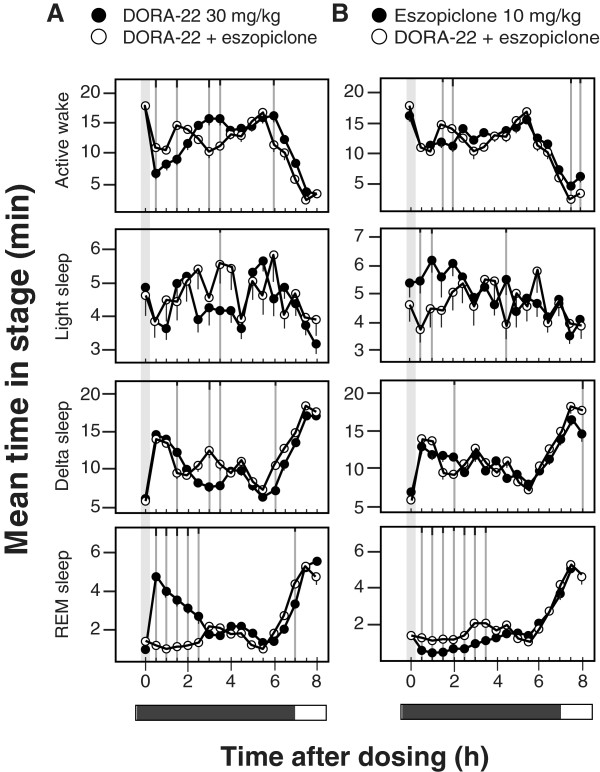
**The combination of DORA-22 and eszopiclone exhibits non-additive effects relative to either agent alone.** Vehicle (vitamin E TPGS, 20% solution, orally), DORA-22 (30 mg/kg), and eszopiclone (10 mg/kg) were dosed alone, or DORA-22 and eszopiclone were administered concomitantly in rats (n = 14) during the active phase (ZT 17:00, gray bar) in the following paradigm: 3 consecutive days of vehicle, 3 days of eszopiclone alone, 3 days of eszopiclone plus DORA-22, and 3 days of DORA-22 alone. Data from all days of each condition were averaged and plotted in 30-min intervals. **A**. Comparison of responses to DORA-22 (closed symbols) relative to the combination of DORA-22 plus eszopiclone (open symbols). **B**. Comparison of responses to eszopiclone (closed symbols) relative to the combination of DORA-22 plus eszopiclone (open symbols). Significant differences at 30-min intervals were determined using a linear mixed-effects model for repeated measures t-test, where significant responses are indicated by gray lines and tic marks indicate significance level (short, medium, long, P < 0.05, 0.01, 0.001).

As these results suggest that orexin and GABA signaling may be largely redundant in their control of active wake and NREM sleep but diverge in their control of REM, the impact of combined administration of DORA-22 (30 mg/kg) and eszopiclone (10 mg/kg) on sleep parameters compared with administration of each agent alone was evaluated in wild-type rats. Relative to DORA-22 alone, the combination exhibited non-additive effects on active-wake reduction (biphasic 1.5 hour increase followed by 1 hour decrease) and only marginal increases in light and delta sleep (Figure 6A). REM sleep, however, was substantially reduced by combination treatment for 2.5 h following treatment. Compared with eszopiclone alone, the combination induced no clear decreases in active wake or increases in delta sleep with the possible exceptions of light and REM sleep (Figure 6B). Transient reductions in light sleep (1 hour) suggested a small influence of DORA-22 to counter the activity of eszopiclone. The combination of DORA-22 and eszopiclone, however, did significantly increased REM sleep relative to eszopiclone alone for up to 3.5 h following treatment. Together these results demonstrate that the pathways underlying the influence of orexin receptor antagonism on sleep parameters overlap with those of the GABA_A_ receptor modulator with the exception of REM sleep, which appears to be mediated by distinct pathways.

## Discussion

These analyses demonstrate that DORAs promote sleep architecture that is indistinguishable from normal resting-phase sleep in animal models, utilizing a mechanism that is distinct from the GABA_A_ receptor modulator, eszopiclone. DORAs have been previously shown to promote NREM and REM sleep proportionately across mammalian species, including man [10–12, 14–16]. Here we demonstrate that the sleep architecture promoted by DORA-12 during both the active and inactive phase closely mimics that seen during the normal resting phase following vehicle treatment, whereas eszopiclone suppresses REM sleep to levels below those seen even during the active phase (see Figure 2). Notably, both NREM and REM sleep induced by DORA-12 at 30 mg/kg is similar in both magnitude and time course to normal inactive-phase sleep. Further, the qEEG changes associated with DORA-12-induced somnolence are also similar to those seen during inactive-phase sleep across frequency bands, the differences being an augmentation of increases in lower frequencies and decreases in higher frequencies typically associated with normal resting-phase sleep. While specific qEEG frequency band definitions (the frequency range assignment [in hertz] of delta, theta, alpha, sigma, beta, and gamma bands) can differ between species and laboratories, it is clear from these studies that the pattern of qEEG changes induced by DORA-12 is similar, if not indistinguishable, from that seen during the resting phase, regardless of band definitions. Eszopiclone responses, on the other hand, exhibit substantial differences from what is typically seen during the resting phase, including dramatically increased beta power, increased gamma power, and decreased lower-frequency alpha, delta, and theta power, the latter potentially being associated with REM suppression. Our qEEG findings corroborate prior evaluations in rats and humans, in which DORA-22 and SB-649868 each minimally disrupted sleep-stage–dependent qEEG spectral power in comparison with the GABA_A_ receptor modulators, which substantially disrupted qEEG spectral power in both the active and inactive phases [13, 14]. In fact, a recently published clinical study demonstrated that suvorexant minimally impacts qEEG spectral density during NREM and REM sleep relative to placebo in both healthy subjects and insomnia patients while trazadone and the GABA_A_ receptor modulators, zolpidem and gaboxadol, induced distinct profiles in human subjects [31].

It has been suggested that the REM promotion by DORAs exceeds what might be expected for normal sleep [32, 33], yet these assertions have been made in studies that lack a specific characterization of the time course and magnitude of REM sleep during the normal resting phase. By superimposing the sleep architecture observed during the inactive phase following vehicle treatment with that occurring during the active phase following vehicle treatment here, we have been able to demonstrate that both NREM and REM sleep increase substantially with the onset of sleep in rats, and that DORA-12 increases both vigilance states in a way that is similar to that seen normally at the onset of the inactive phase (see Figure 2; Additional file 1: Figure S1). Further, it is unclear what detrimental effects may result from normal or moderate increases in REM sleep during the inactive phase, and any causal relationships with behavior have yet to be demonstrated. On the other hand, it is clear that REM deprivation is associated with physiological and cognitive deficits. In rats, REM sleep deprivation by the disk-over-water method is associated with a severe phenotype characterized by weight loss with paradoxical hyperphagia, impaired thermoregulation, and eventual fatality [18, 34, 35]. Selective REM deprivation has also been shown to have hyperalgesic effects in sleep laboratory studies [24]. Perhaps the most well-studied effects are on impairments in learning and memory in rats; REM deprivation impairs hippocampus-dependent spatial learning, and is associated with molecular and cellular alterations in hippocampal function [19, 20, 36–38]. In fact, a recent study examining the effects of zolpidem in human subjects found that deficits in memory improve the day following administration, a time at which drug levels were expected to have diminished, interpreted to suggest that the quality of sleep induced by this GABA_A_ receptor modulator affects cognitive performance [39].

On the other hand, our REM deprivation studies in mice demonstrate that DORA-22 effectively promotes both delta and REM sleep immediately upon transfer from deprivation cages to those containing normal bedding, a recovery that was more immediate than in untreated control mice. In this paradigm, transfer to a normal cage represents a novel environment such that untreated control mice continue to exhibit arousal for up to an hour. Both DORA-22 and eszopiclone significantly attenuated active-wake phase immediately upon transfer to the recovery condition, but DORA-22 facilitated REM recovery while eszopiclone actually suppressed REM sleep at later time points relative to vehicle despite a presumed homeostatic drive for REM recovery. In untreated control mice, delta sleep increased with a time course that preceded REM-sleep increases, while DORA-22 increased REM nearly coincident with delta sleep, suggesting that DORAs may facilitate recovery in response to homeostatic drive. Indeed, it has been suggested that the OX_1_R antagonism provided by DORAs may allow for disinhibition of REM sleep [33], which may underlie yet another favorable property of DORAs in their ability to respond to accumulated REM sleep debt. If this is indeed the case, variability in sleep architecture, including the magnitude and timing of REM sleep promotion observed in animal models at different laboratories, would be expected to be a function of the prior housing conditions; differences in total sleep debt or specific sleep stage debt in EEG-implanted mice or rats would be expected to give rise to differences in responses to DORA treatment. Further study is required to determine the ability of both ORAs and GABA_A_ receptor modulators to respond to homeostatic needs and the behavioral and psychiatric consequences resulting from those manipulations.

The hypnotics compared herein – GABA_A_ receptor modulators and DORAs – promote sleep via distinct, but overlapping mechanisms of action. Orexin neurons and their cognate receptors are more discretely distributed in the brain relative to GABA_A_ receptor subtypes, and targeting orexin-mediated arousal with ORAs has more restricted effects relative to the GABA_A_ receptor modulators, which augment GABA_A_ receptor activity, resulting in widespread central nervous system depression. Within the sleep system, orexin neuron activity promotes arousal and vigilance-state control through their projections to tuberomammillary nuclei and brain stem structures including the dorsal raphe, locus coeruleus, and laterodorsal and pedunculopontine tegmental nuclei, while inhibitory GABAergic projections from ventrolateral preoptic nuclei project to many of the same structures as well as orexin neurons [40]. However, the widespread distribution and function of GABA_A_ receptor subtypes involved in other complex behaviors—including, but not limited to anxiety, locomotor coordination, addiction, learning, and memory [41, 42] —may underlie the differential sleep-promoting effects of GABA_A_ receptor modulators across species (e.g., sleep promotion in rats, monkeys, and humans, but paradoxical hyperarousal in dogs). Species-dependent differences in signal strength mediated by the multitude of GABAergic pathways within and/or outside of the specific sleep pathways are likely responsible. Nevertheless, our findings indicate overlap between orexin and GABAergic pathways, as might be predicted from histological evidence. The sleep effects of DORA-22 were markedly attenuated in orexin neuron─deficient rats, indicating that these effects were mediated through the orexin pathway with some remaining effects likely due to incomplete ablation of orexin-containing neurons in these animals [30]. The selectivity of DORA-22 has previously been demonstrated at doses as high as 100 mg/kg in mice lacking both orexin receptors, where no detectable effects were seen [12]; as such, the residual REM promotion seen in *Ox/Atx* transgenic rats is likely due to residual orexin signaling in those animals. The effect of eszopiclone on active wake and NREM sleep was also attenuated in rats lacking orexin neurons, and the combined administration of both agents had similar effects on active wake compared with either administered alone. These results suggest that both classes of compounds reduce active wake through a common pathway. REM sleep, however, was similarly suppressed by eszopiclone in both *Ox/Atx* transgenic rats and in wild-type control rats. The combined administration of eszopiclone with DORA-22 markedly reduced REM relative to DORA-22 alone, and only slightly increased REM relative to eszopiclone alone, indicating that these effects on REM sleep are not entirely additive. Both orexinergic and GABAergic signaling influence the activity of brainstem nuclei, particularly the locus coeruleus, to affect vigilance-state control, including REM sleep. These results indicate that these influences are not entirely dependent on one another, but can influence this function through parallel means. From a therapeutic standpoint, these overlapping, yet distinct mechanisms have important implications regarding tolerance and dependence. GABA_A_ receptor modulators have been shown in both animal models and human subjects to exhibit tolerance to repetitive dosing, becoming less efficacious over time [43–45], while ORAs, including suvorexant (Belsomra®), have shown no tolerance or evidence of withdrawal even after a year of treatment in patients [17] and no diminution of efficacy in animal models (reviewed in [46]). While the role of orexin in reward and withdrawal suggest that ORAs may have the capacity to diminish the dependence on rewarding influences (reviewed in [47]), the effectiveness of ORAs in animal models and human subjects following treatment with the current standard of care, GABA_A_ receptor modulators, remains to be determined.

## Conclusions

While no major differences in PSG and qEEG profiles were observed during DORA-induced sleep in this study, eszopiclone had marked effects on PSG and qEEG profiles, suggesting that GABA_A_ receptor modulators may have effects that do not resemble natural sleep or qEEG patterns. The observed effects of eszopiclone on REM sleep, but not active wake and NREM sleep in *OX/Atx* mice or in the presence of co-administered DORA-22, support the idea that eszopiclone and DORA-22 impact REM sleep via divergent pathways but may share a common (orexin-dependent) mechanism for their influence on active wake and NREM sleep. The potential for ORAs to promote sleep in a way that is qualitatively similar to natural sleep suggests that orexin receptor antagonists may have promise as a novel therapeutic for insomnia.

## Methods

### Animals and compound administration

All animal experiments were performed in accordance with The National Research Council’s Guide for the Care and Use of Laboratory Animals ( http://www.nap.edu/catalog.php?record_id=12910) and were approved by the Merck Institutional Animal Care and Use Committee. All efforts were made to minimize animal use and suffering. The animals were singly housed with food and water available ad libitum, and on a 12:12 light:dark cycle. All compounds were administered orally at the indicated dosages in vitamin E TPGS, 20% solution.

In adult male C57/BL6NTac wild-type mice (ages 9 to 14 weeks; Taconic Farms, Germantown, NY), DORA-22 (100 mg/kg), eszopiclone (60 mg/kg), or vehicle were administered during the active (dark) phase 4 h prior to lights-on (ZT 20:00, where ZT 00:00 is lights-on). Treatments were administered for 5 days in a balanced crossover design (5 days of compound or vehicle, followed by a 2-day washout and 5 days of conditional crossover), and the compound and vehicle conditions for each animal were combined and averaged over a 24-h time period before determining the effects relative to vehicle.

Adult male Sprague–Dawley wild-type rats (c.a. 600 g; 6–12 months of age; Taconic Farms, Germantown NY) were treated with DORA-22 (30 mg/kg), DORA-12 (30 mg/kg), eszopiclone (10 mg/kg), or vehicle during the active (dark) phase (7 h prior to lights-on, ZT 17:00) or inactive (light) phase (1 h prior to lights-off, ZT 23:00) in a balanced crossover design (1–2 days of vehicle run-in [all], 3 days on vehicle or compound treatment, 2–3 days of washout, 3 days on reverse arm). The orexin/ataxin-3 (*Ox/Atx*) rats, which exhibit postnatal loss of orexin neurons, have been described elsewhere [48] and were licensed from the University of Texas Southwestern Medical Center and maintained at Taconic Farms, Germantown NY.

Adult male beagles (9–17 kg; Marshall BioResources, North Rose, NY) were treated with DORA-22 (3 mg/kg) or eszopiclone (5 mg/kg) during the active phase 9 h prior to lights-off (ZT 03:00). Cognitive testing was performed 2.5 to 3 h following DORA-22 treatment, but found no differences from vehicle in PSG or qEEG recordings (not shown). For PSG analysis, a block repeated-measured design was employed, in which all dogs received vehicle for 5 days, followed by a 2-day washout and 5 consecutive days of DORA-22 treatment.

Adult male rhesus monkeys (*Macaca mulatta*, 6.9 - 13 kg; The Mannheimer Foundation, Homestead, FL, Covance Research Products, Denver, PA, and the University of Louisiana at Lafayette, Lafayette, LA) were treated with DORA-22 (30 mg/kg), eszopiclone (10 mg/kg), or vehicle during their active phase (6.5 h prior to lights-off, ZT 05:30). A 1-day block crossover design was used, in which all subjects received 1 day of vehicle and 1 day of compound treatment.

### Sleep architecture and qEEG recordings

The durations of sleep stages were quantitated by PSG in mice and rats subcutaneously implanted with radio telemetric physiologic monitors (Data Sciences International, Arden Hills, MN) to simultaneously record continuous electrocorticogram (ECoG) and electromyogram (EMG) activities, as previously described [11, 12]. Polysomnography was performed in telemetry-implanted dogs and rhesus monkeys via ECoG, EMG, and electrooculogram (EOG), as described previously [11, 49]. Sleep scoring and methods for determining differences in the amount of time spent in active wake and various sleep stages (light, delta, and REM sleep in mice and rats; NREM I/SWS I, NREM II/SWS II, and REM in dogs and rhesus monkeys) has been described in detail elsewhere [11, 13, 50].

Quantitative EEG scoring was performed on ECoG data collected during sleep experiments from telemetry-implanted C57/BL6 mice, Sprague–Dawley rats, beagles, and rhesus monkeys with modifications to that previously described [13]. Briefly, spectral analysis of continuous EEG was quantified for vehicle and compound conditions after scoring continuous frequencies into canonical frequency bands (delta, 0.5–4 Hz; theta, 4.0–7.0 Hz; alpha, 8.0–12 Hz; sigma, 12–16 Hz; beta, 19–30 Hz; and gamma, 35.0–100.0 Hz). Quantitative EEG values are spectral power (uV2) log transformed before analysis and averaged over 30-min intervals. Results are expressed as means ± standard error of the mean (SEM). Comparisons with vehicle were performed using a mixed-model analysis of variance (ANOVA) at each time point with random effects for subject and date within subject in the R statistical computing environment (cran.us.r-project.org; the R Foundation for Statistical Computing, Vienna, Austria). A linear mixed-effects model was used for significance testing. Significant differences between conditions at 30-min intervals are indicated in the corresponding figures with gray vertical lines through significantly different data points and tic marks indicating significance level (short, medium, long, P < 0.05, 0.01, 0.001).

### REM deprivation and recovery in mice

REM deprivation in mice was performed using modifications to the "flower pot" method previously described [29]. Cages were prepared that contained four cylindrical pedestals (3 cm in diameter × 2.5 cm tall) fixed to the bottom of the enclosure, roughly equidistant from one another, allowing mice to move from one location to another within the cage to access food and water. Under deprivation conditions, water was added to the bottom of the cage to a height of approximately 2 cm such that REM sleep - induced atonia was associated with water exposure and immediate arousal. Control conditions included cages containing pedestals without water. In all experiments, the deprivation (or no water control) condition proceeded for 28 h beginning at ZT 20:00 (late active phase) to ZT 24:00/00:00 (inactive-phase onset) on the subsequent day at which time the animals were returned to cages containing normal Bed o’ Cobb bedding to allow recovery sleep. All experiments were separated by at least 3 days of normal bedding conditions.

REM deprivation and recovery in the absence and presence of pharmacological treatment was performed in telemetry-implanted mice in which vigilance state was evaluated continuously (PSG described above). Experiments utilized a 2 × 3 day balanced crossover design in which each animal experienced both the control and experimental conditions on alternative arms of the experiment. In baseline studies measuring recovery after REM deprivation, the first arm of these experiments was initiated with 28 h of deprivation or control conditions (pedestal cages with or without water, respectively), followed by 44 h of recovery (28 + 44 = 72 h, or 3 days), and continued with a second 28-h deprivation and 44-h recovery period. After an additional day of washout, the second arm of the study proceeded identically to the first except that the experimental and control groups were reversed such that each animal received both treatments. In experiments evaluating the impact of DORA-22 and eszopiclone on REM recovery, all animals experienced deprivation for 28-h periods followed by REM recovery, at which time vehicle and drug (DORA-22 or eszopiclone) were administered in the 2 × 3 day balanced crossover design described above. REM recovery was evaluated in 10-min intervals following return to cages containing normal bedding.

### Statistical analyses

Statistical comparison of control and experimental conditions at individual time points in PSG and qEEG experiments (Figures 1, 3, 4, 5 and 6) was determined using a linear mixed-effects statistical model with repeated measures analysis of variance (ANOVA) applied using the R statistical software application (cran.us.r-project.org; the R Foundation for Statistical Computing, Vienna, Austria, v3.0.1; nlme package v3.1-111) with fixed effects for treatment and random effects for subject at each time point to identify points of statistical significance. Biological/pharmacological significance was subsequently determined by consecutive significance points (P < 0.05) with similar sequential trend. Gray vertical lines indicate treatment differences of p < 0.05 with vertical black segment lines indicating level of significance (short: P < 0.05; medium: P < 0.01; long: P < 0.001). Significant differences in responses between multiple conditions over a defined time period (150 min for the experiments in Figure 2) were determined in the R statistical software application by two different methods and are reported separately. The first evaluated data combined from all four conditions by repeated measures ANOVA followed by Tukey multiple comparison (HSD) test to determine significant differences between each condition. A second method compared individual conditions in a pairwise manner by repeated measures ANOVA to determine the F statistic and P value for each of six comparisons between four conditions.

## Electronic supplementary material

Additional file 1: Figure S1: Showing detailed sleep architecture comparisons between active- and inactive-phase sleep following vehicle treatment, as well as active- and inactive-phase sleep architecture following treatment with DORA-12 (30 mg/kg) and eszopiclone (10 mg/kg), is supplied as supporting data to that summarized in Figure 2. (PDF 3 MB)

## Abbreviations

DORA: Dual orexin receptor antagonist
EEG: Electroencephalography
qEEG: Quantitative electroencephalography
ECoG: Electrocorticogram
EMG: Electromyogram
EOG: Electrooculogram
GABA_A_: Gamma-aminobutyric acid A receptor
NREM: Non-rapid eye movement
OX_1_R: Orexin 1 receptor
OX_2_R: Orexin 2 receptor
ORA: Orexin receptor antagonist
*Ox/Atx*: Transgene containing cytotoxic *poly-Q-ataxin-3* gene product driven by the human *hypocretin* [*HCRT*] gene promoter
p.o.: Per os, or oral administration
PSG: Polysomnography
REM: Rapid eye movement
TMN: Tuberomammillary nuclei
TPGS: d-alpha-tocopheryl polyethylene glycol 1000 succinate
VLPO: Ventrolateral preoptic nucleus
ZT: Zeitgeber time.
