# Supplementary material for: Differential sleep-promoting effects of dual orexin receptor antagonists and GABAA receptor modulators
Source: BMC Neurosci. 2014 Sep 22;15:109. doi: 10.1186/1471-2202-15-109 (PMC4261741; doi:10.1186/1471-2202-15-109)
Supplement: Supplementary file 1 — Additional file 1: Figure S1: Showing detailed sleep architecture comparisons between active- and inactive-phase sleep following vehicle treatment, as well as active- and inactive-phase sleep architecture following treatment with DORA-12 (30 mg/kg) and eszopiclone (10 mg/kg), is supplied as supporting data to that summarized in Figure 2. (PDF 3 MB) [file 12868_2014_3800_MOESM1_ESM.pdf]

Supplemental Figure 1. Sleep-promoting effects of DORA-12 were attenuated during the normal resting phase, unlike eszopiclone, in rats. Active-phase treatment occurred at ZT (Zeitgeber time) 17:30 (6.5 h prior to lights-on) and inactive- phase treatments at ZT 23:00 (1 h prior to lights-on). Mean time in specific sleep stages during 30-min analysis intervals following treatment (gray bars) are shown. Control and experimental conditions are shown in filled and open symbols, respectively. **A.** Comparison of inactive-phase and active-phase sleep architecture following vehicle treatment. Vehicle conditions from both active- and inactive-phase DORA-12 and eszopiclone studies were separately combined and plotted on the same time course such that treatment times were coincident (6.5 h shift of PSG data). **B.** Active-phase treatment of DORA-12 (30 mg/kg, n=14) and eszopiclone (10 mg/kg, n=16) relative to vehicle (vitamin E TPGS, 20% solution, orally). **C.** PSG responses to DORA-12 (n=7) and eszopiclone (n=8) following treatment 1 h prior to inactive-phase onset. Results of experiments B and C were the result of 3-day balanced crossovers in which each animal served in both control and experimental conditions. Significant differences between conditions at each time point are indicated by gray vertical lines, and black tic marks indicate significance level (short, medium, long, $P < 0.05, 0.01, 0.001$).

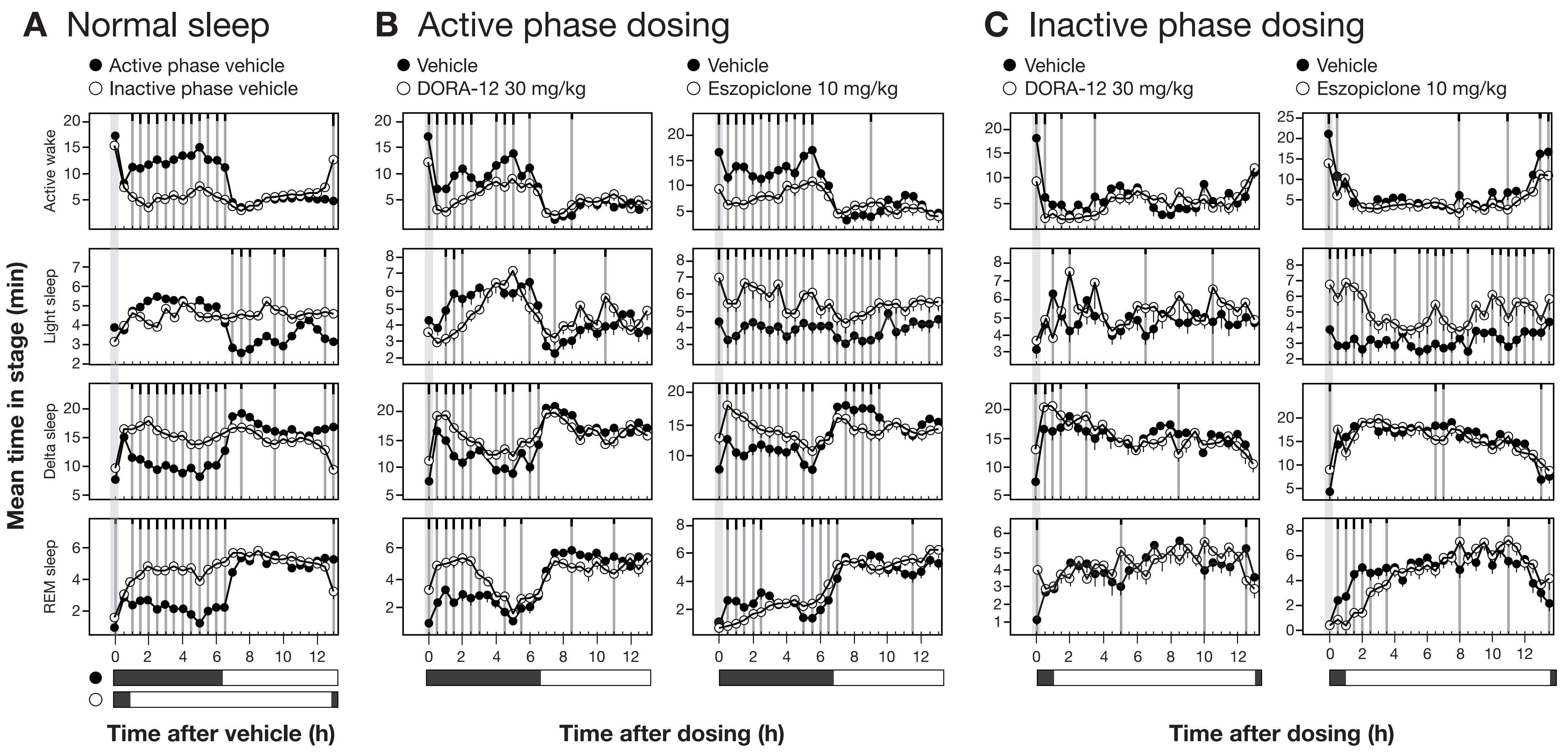
A Normal sleep
Active phase vehicle
Inactive phase vehicle
B Active phase dosing
Vehicle
DORA-12 30 mg/kg
Vehicle
Eszopiclone 10 mg/kg
C Inactive phase dosing
Vehicle
DORA-12 30 mg/kg
Vehicle
Eszopiclone 10 mg/kg
Mean time in stage (min)
Active wake
Light sleep
Delta sleep
REM sleep
Time after vehicle (h)
Time after dosing (h)
Time after dosing (h)
